# Machine learning analysis of gene expression profile reveals a novel diagnostic signature for osteoporosis

**DOI:** 10.1186/s13018-021-02329-1

**Published:** 2021-03-15

**Authors:** Xinlei Chen, Guangping Liu, Shuxiang Wang, Haiyang Zhang, Peng Xue

**Affiliations:** Department of Orthopedics, Zibo Central Hospital, Zibo, 255000 Shandong China

**Keywords:** Osteoporosis, WGCNA analysis, PPI network, Logistic regression model

## Abstract

**Background:**

Osteoporosis (OP) is increasingly prevalent with the aging of the world population. It is urgent to identify efficient diagnostic signatures for the clinical application.

**Method:**

We downloaded the mRNA profile of 90 peripheral blood samples with or without OP from GEO database (Number: GSE152073). Weighted gene co-expression network analysis (WGCNA) was used to reveal the correlation among genes in all samples. GO term and KEGG pathway enrichment analysis was performed via the *clusterProfiler* R package. STRING database was applied to screen the interaction pairs among proteins. Protein–protein interaction (PPI) network was visualized based on Cytoscape, and the key genes were screened using the cytoHubba plug-in. The diagnostic model based on these key genes was constructed, and 5-fold cross validation method was applied to evaluate its reliability.

**Results:**

A gene module consisted of 176 genes predicted to be associated with the occurrence of OP was identified. A total of 16 significantly enriched GO terms and 1 significantly enriched KEGG pathway were obtained based on the 176 genes. The top 50 key genes in the PPI network were identified. Then 22 genes were screened based on stepwise regression analysis from the 50 key genes. Of which, 9 genes were further screened out by multivariate regression analysis with the significant threshold of *P* value < 0.01. The diagnostic model was established based on the optimal 9 key genes, which efficiently separated the normal samples and OP samples.

**Conclusion:**

A diagnostic model established based on nine key genes could reliably separate OP patients from healthy subjects, which provided novel lightings on the diagnostic research of OP.

**Supplementary Information:**

The online version contains supplementary material available at 10.1186/s13018-021-02329-1.

## Introduction

Osteoporosis (OP) is defined as a systemic skeletal disease which is characterized by low bone mass and micro-architectural deterioration of bone tissue, and even can lead to an increasing risk in bone fracture [1, 2]. According to the Surgeon General’s report on bone health and OP, OP has affected more than 8 million women and 2 million men in the USA, as well as approximately 34 million people undergo low bone mass [3]. Because the early symptoms of OP are not obvious or even asymptomatic, early diagnosis and timely intervention can certainly prevent the disease from progressing to a more serious direction [4, 5]. Although, there are numbers of OP biomarkers and laboratory techniques available, each of which has its own strength and limitation [6]. Therefore, the identification and development of specific and sensitive biomarkers for OP diagnosis or treatment is more urgent.

Recently, a series of OP-related genes that have been identified can act as potential diagnostic biomarkers and may potentially be applied for the clinical diagnosis for OP. For example, Zhang et al. analyzed the correlation between VDR gene polymorphisms and OP risk as well as bone mineral density (BMD) in postmenopausal women through a systematic meta-analysis and suggested that VDR is susceptible to OP and might be a potential biomarker [7]. Qian et al. found that PPWD1 may be considered as a potential diagnostic biomarker for the occurrence of postmenopausal OP based on the weighted gene co-expression network analysis (WGCNA) [8]. Mondockova et al. revealed that the estrogen receptor 1 gene can regulate BMD, and the treatment efficiency of OP in Slovak postmenopausal women may more likely be a biomarker [9]. In addition, many non-coding RNAs such as lncRNAs and circRNAs may also play an essential role in OP progression and also can be promising biomarkers [10]. LncRNA-NEF is significantly downregulated in postmenopausal OP and is closely associated with the course of treatment and recurrence [11]. Chen et al. have identified a group of circulating miRNAs as non-invasive biomarkers for the detection of postmenopausal and mechanical unloading OP through a large-scale screening based on microarray [12]. However, model analysis, always in the presence of other covariates, has been widely used to improve the probability of prediction compared with that of single genes [13, 14]. Despite of a large number of single biomarkers identified through different ways, the model analysis based on multiple key genes in OP is still lacking.

In the present study, a reliable diagnostic model was established based on nine key genes which might be involved in the progression of OP, and could efficiently separate normal subjects from OP patients. Our study enriched the research of early diagnosis in OP and exerted certain practical values.

## Materials and methods

### Data collection

The mRNA profile GSE152073 was downloaded from the Gene Expression Omnibus (GEO, https://www.ncbi.nlm.nih.gov/geo/) which includes 90 peripheral blood samples of 44 OP patients and 46 healthy subjects. The mRNA profiles of GSE152073 were detected by using Affymetrix Human Transcriptome Array 2.0.

### Weighted gene co-expression network analysis

“*WGCNA*” function package of R language as previously described [15]. Briefly, the hierarchical clustering of genes was conducted based on the gene expression value of targeted genes, and genes with high expression similarity were placed into the same module by using the dynamic shear tree method. Next, we calculated the Module Eigengene (ME) of each module and the correlation coefficient between the ME and the phenotype we focused on. In which phenotypes contained disease states (dichotomous phenotypes) (i.e., OP and normal). Besides, age, height, and weight were also included as continuous phenotypes in ME and phenotype correlation analysis. *P* value < 0.05 was used as the significant threshold of ME and phenotype correlation analysis. The greater the absolute value of ME, the closer the correlation between this module and the concerned phenotype.

### Functional enrichment analysis

For these genes in the key modules, the Gene Ontology (GO) term (including biological process, molecular function, and cellular component) and Kyoto Encyclopedia of Genes and Genomes (KEGG) pathway enrichment analysis were performed based on the *clusterProfiler* function package of R language [16] to identify key pathways involved in the progression of OP. *P* < 0.05 was used as the threshold for statistical significance.

### The construction of protein–protein interaction (PPI) networks

The STRING (https://string-db.org/, version 11.0) is a widely used database to analyze and predict the functional connections and interactions of proteins in human diseases [17]. In the present study, STRING was applied for the functional connections and interactions analysis between proteins during the progression of OP, and the interaction pairs with confidence score ≥ 0.4. Subsequently, the PPI network was visualized based on Cytoscape software (https://cytoscape.org/, version 3.7.2) as previously reported [18], and key genes of the PPI network were screened by using the cytoHubba plug-in of Cytoscape software according to the Maximum neighborhood component (MNC) algorithm.

### The construction of logistic regression model

Logistic regression is a widely used method in classification as previously described [19], which is always used to predict a classification, in this study, OP or not. In the present study, all 90 samples were divided into the training set by simple random sampling method and the validation set for the construction of logistic regression model. Here, based on the normal control samples and OP samples, stepwise regression analysis was first used to prioritize genes followed by multivariate logistic regression analysis to determine the final genes contained in the model by using the *glm* function of R language [20]. Of which, the genes’ expression values were used as the continuous variable, and the sample type was considered as the binary classification response value. Meanwhile, the 5-fold cross validation method was used to verify the accuracy of the logistic regression model.

## Results

### The green module was determined to be closely related to the occurrence of OP through WGCNA analysis

**Fig. 1:**
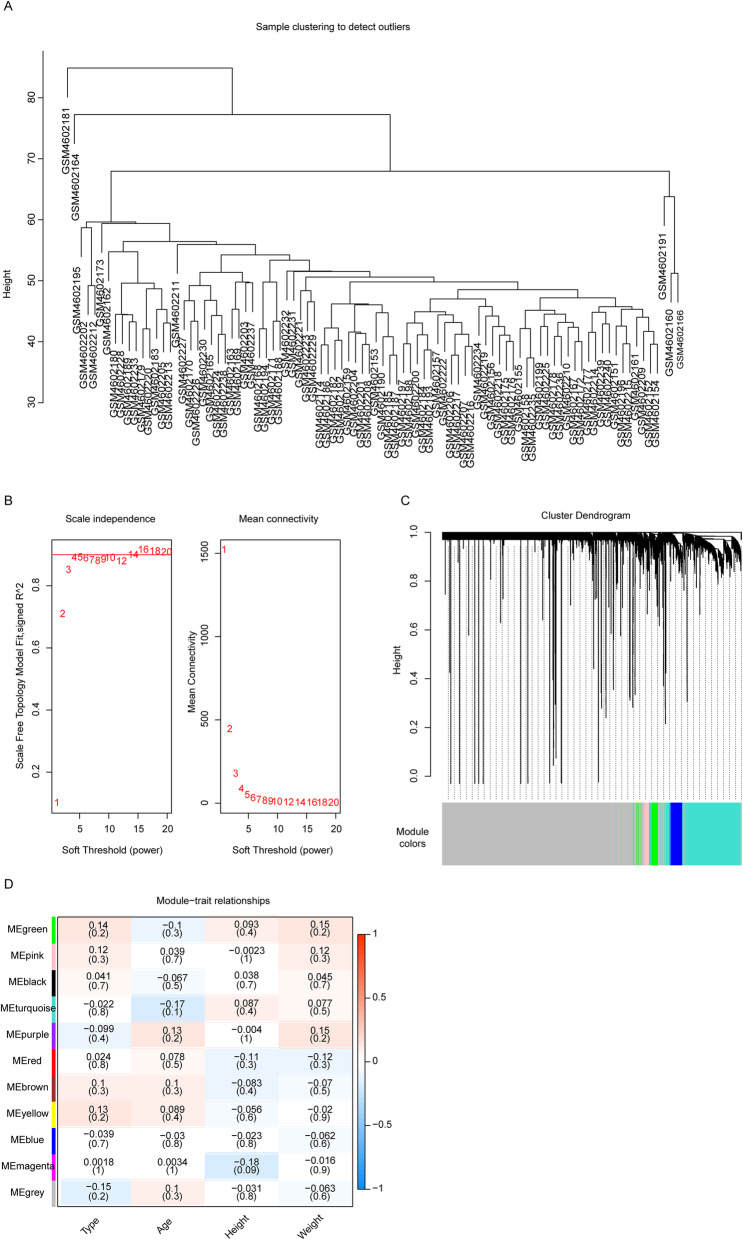
Weighted gene co-expression network analysis (WGCNA). **a** Clustering of the 90 samples. **b** Selection of the soft threshold in the WGCNA. The red line represents the correlation coefficient, and the first point above the red line is the soft threshold (*β* = 14). **c** Clustering of the gene modules. Each designated color represents a gene module, and the genes that could not be grouped into other modules were placed in the grey module. **d** The correlation between gene modules and phenotypes. Darker color indicated greater the correlation between gene modules and phenotypes

Firstly, we standardized the expression data of GSE152073 in order to remove the batch effects, and after standardization, no obvious deviation among all samples was observed. (Fig. S1). Therefore, the standardized data could be adopted for the subsequent analysis. Then the consensus clustering analysis based on the mRNA expression values of 90 samples indicated that there were two outliers (GSM4602181 and GSM4602164), which were excluded in the subsequent WGCNA analysis to ensure the reliability of our analysis (Fig. 1a). Previous studies have demonstrated that the co-expression network conformed to the characteristic of unsigned network [21, 22]. Hence, the soft threshold was selected as 14 to ensure that the co-expression network was unsigned, (Fig. 1b). Then the average-linkage hierarchical clustering method was applied to cluster the genes with setting the minimum cardinality of each gene module to 50 based on the criterion of mixed dynamic shear tree, and Module Eigengene (ME) for each module was then calculated. The cluster analysis on the modules was conducted, and the modules which were close to each other were merged into a new module with setting height 0.25. The results suggested that ten modules were obtained, and the grey module contained the gene set that could not be classified into any module (Fig. 1c). In addition, based on the ME of each module, we also calculated the correlation between each module with sample type as well as race information based on the ME of each module, and found that the green module which contained 176 genes had the greatest correlation with the sample type (Fig. 1d, correlation coefficient = 0.14). These results indicated that the genes in the green module were probably related to the initiation of OP.

### Functional enrichment analysis of potential key genes in the green module

**Fig. 2:**
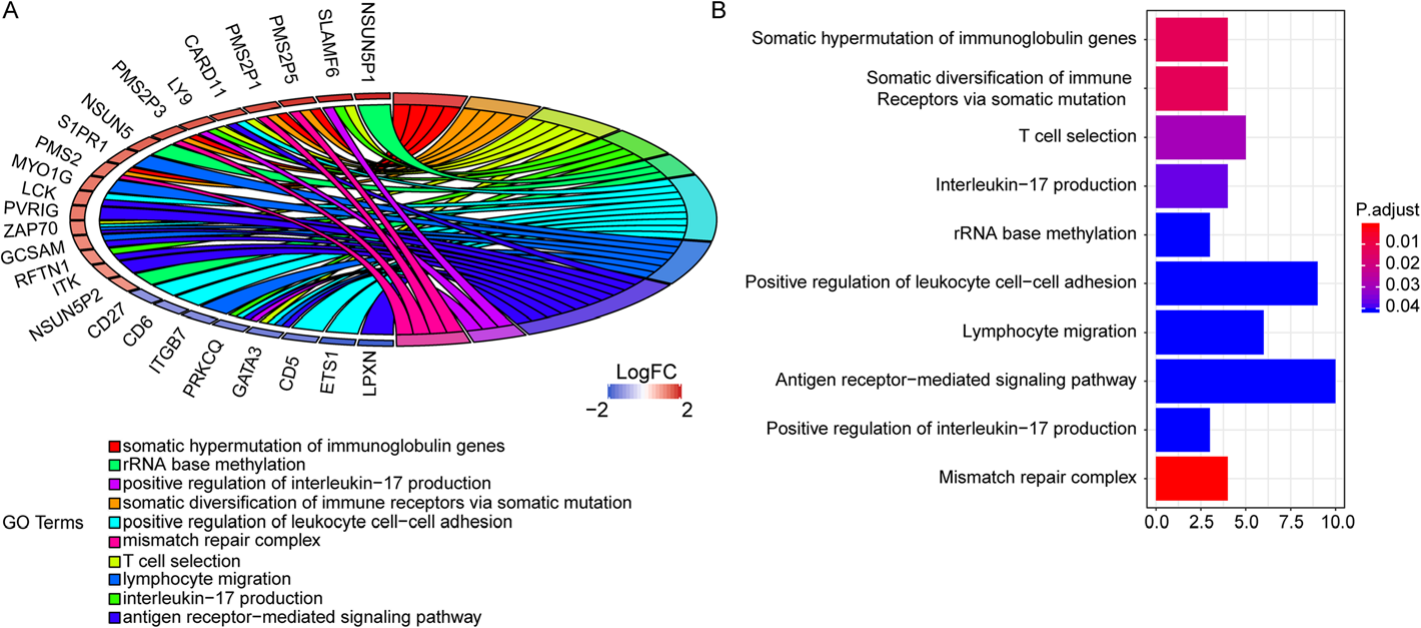
The functional enrichment analysis. **a** The chord diagram of top 10 significantly enriched GO terms. The right semicircle represents the names of 10 GO terms, and the left semicircle are mRNAs in GO terms. **b** The histogram of top 10 significantly enriched GO terms. The horizontal axis represents the number of genes, and the vertical axis represents the name of GO terms

The GO term and KEGG pathway enrichment analysis were then performed to identify important pathways involved in the development of OP based on the 176 genes. The analysis identified 16 significantly enriched GO terms including somatic hypermutation of immunoglobulin genes, interleukin-17 production, and positive regulation of leukocyte cell–cell adhesion as well as one significantly enriched KEGG pathway (T cell receptor signaling pathway). Specifically, the top 10 most significantly enriched GO terms are shown in Fig. 2a and b. All of the significantly enriched GO terms and KEGG pathways are provided in Table S1. These results suggested that these enriched GO terms and KEGG pathways might be associated with OP progression.

### Top 50 key genes in the PPI network were identified

**Fig. 3:**
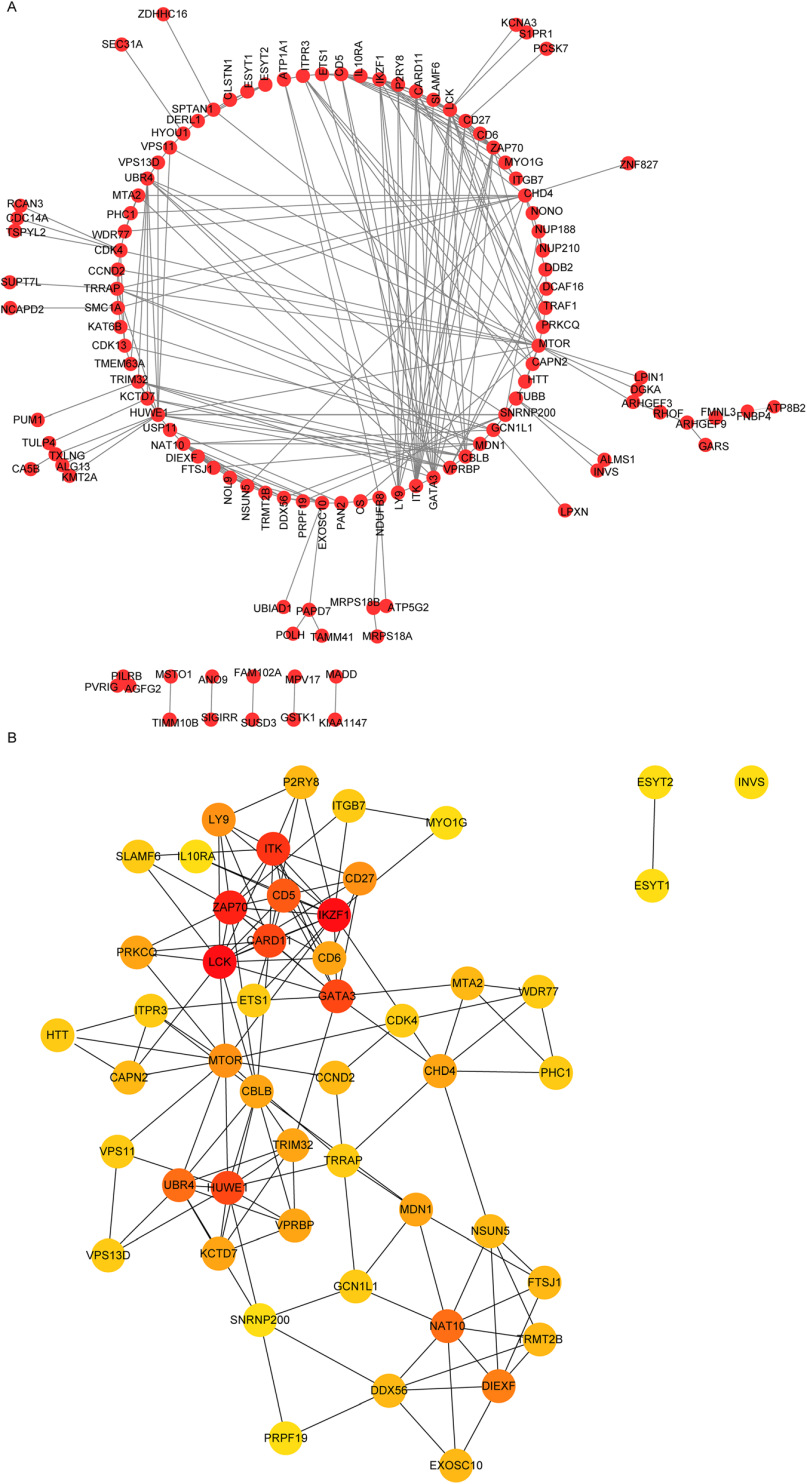
The construction of PPI network. **a** The PPI network. Each dot represents a node. The more lines connected to the dot, the higher the degree of this node, which means the more important the genes on this node may be in the network. **b** The top 50 genes with high degree in PPI network were screened based on MNC algorithm. The darker the red color was, the higher the degree was

The PPI network was visualized by using the 176 genes in the green module and shown in Fig. 3a. The PPI network analysis based on the 176 genes in the green module showed that there were 119 nodes and 224 edges, of which, a node is a gene and the edge represents the interaction between them. More importantly, the top 50 genes were screened after sorting by score obtained using MNC algorithm (Fig. 3b), and these genes were considered as the potential key genes for OP (Table S2).

### The diagnostic model could reliably separate OP patients from healthy subjects

**Fig. 4:**
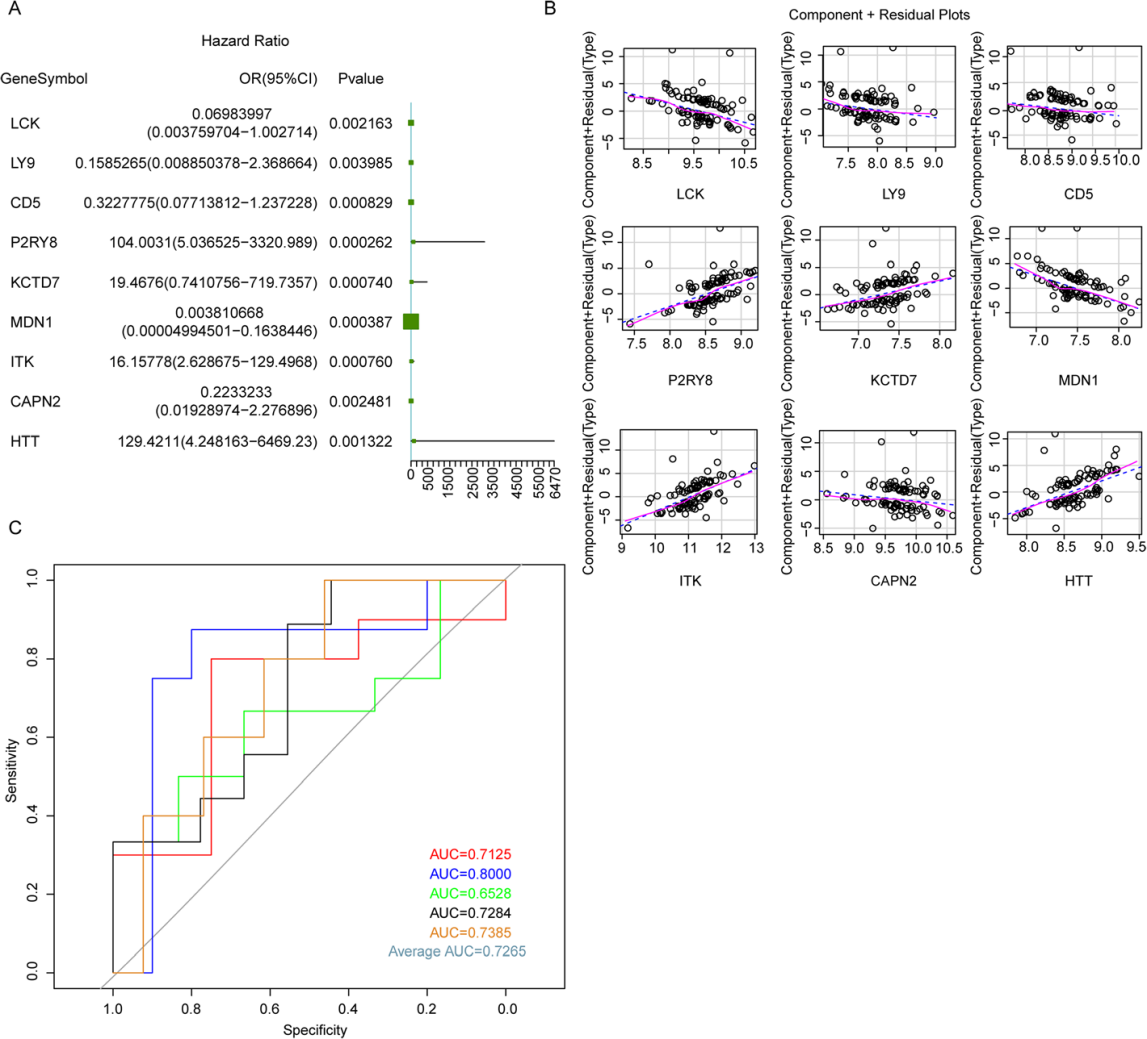
The construction of logistic regression diagnostic model. **a** The forest map of 9 optimal mRNAs in logistic regression model. *P* < 0.01 demonstrated that the mRNA contributes more to the model. **b** The component plus residual plot of 9 mRNAs in logistic regression model. The obvious linear relationship between the horizontal axis and the vertical axis indicates that the independent variables are suitable to be brought in the model. **c** The ROC curve of model. The horizontal axis represents false-positive rate, and the vertical axis represents true-positive rate. AUC value could assess the accuracy of the model, and the greater AUC value indicates higher accuracy of the model

The stepwise regression analysis was first performed, which screened out 22 genes from the 50 key genes. Multivariate logistic regression analysis further identified 9 genes, including LCK, LY9, CD5, P2RY8, KCTD7, MDN1, ITK, CAPN2, and HTT, that are still significant (*P* < 0.01) in multivariate logistic regression analysis (Fig. 4a), suggesting that these 9 genes might be significantly related to the occurrence of OP. Finally, the logistic regression model was constructed based on the 9 key genes, and we found that the model obeys the normal distribution (Fig. S2A). Meanwhile, we found that the independent variables in the model all had significant linear correlation with the response variables (Fig. 4b), and no extreme points that could influence the accuracy of the model were observed (Fig. S2B). All those data should illustrate the robustness of the model in OP occurrence prediction.

Further, the reliability and validity of this model were evaluated by 5-fold cross validation method. The AUC value of the 5 logistic models in the 5 validation sets was 0.7125, 0.8000, 0.6528, 0.7284, and 0.7385, respectively, with the average AUC 0.7265 (Fig. 4c). Besides, C-index of the model was 0.781, indicating that the model we established had a good consistency with the actual situation. These data suggested that the logistic regression model established by bringing into the 9 optimal genes could reliably distinguish the type of samples (OP or not).

## Discussion

According to the World Health Organization (WHO), OP has become one of the most common diseases, and 30–50% of all women worldwide will undergo fractures because of OP throughout their lives [23]. On the other hand, since OP is clinically symptomless until the first fracture occurs, early diagnosis is more critical and contributes to timely intervene OP to relieve the patient’s pain [24]. In this study, we identified 176 genes that might be related to the occurrence and development of OP through WGCNA analysis. Meanwhile, the functional enrichment analysis was performed based on the 176 genes, and the results indicated that several GO terms such as somatic hypermutation of immunoglobulin, interleukin-17 production, and positive regulation of leukocyte cell–cell adhesion as well as only one KEGG pathway T cell receptor signaling pathway were significantly enriched. Previous studies have revealed that both the bone and immune systems work as a close-knit functional unit (osteoimmune system) due to their common developmental niche [25]. McInnes et al. demonstrated that dysregulation of the immune system has already been involved in the initiation of various inflammatory autoimmune diseases resulting in adverse effects on bone integrity including OP [26]. In addition, T cells are identified to participate in the pathological bone loss associated with a series of conditions including ovariectomy-induced bone loss [27], rheumatoid arthritis [28], and bisphosphonate-associated osteonecrosis of the jaw [29]. Our analysis indicated that a total of 176 genes might be closely associated with the development of OP, which consists of previous studies that immune response occurs in the progression of OP. These results further provided a certain theoretical basis for our predictive model.

Then we identified the top 50 genes that were predicted to be closely related to the development of OP according to the score based on the PPI network. To establish a more explanatory model, the logistic regression analysis was performed and screened 9 optimal genes (LCK, LY9, CD5, P2RY8, KCTD7, MDN1, ITK, CAPN2, and HTT) (*P* < 0.01) which contributed more to the model. Lewis et al. describe a disorder in bone homeostasis in transgenic mice that anomalously express the cytokine interleukin 4 (IL-4) under the direction of the lymphocyte-specific proximal promoter for the LCK gene, and found that bone disease in LCK-defective mice appeared to because of markedly decreased bone formation [30]. Sundberg et al. performed a flow cytometric analysis of peripheral blood lymphocytes in OVX rats revealed that both CD5 and CD4 positive lymphocytes or the mean fluorescence per cell are all increased [31]. One previous study reported that the potentiation of 5-HT signaling through the inhibition of the 5-HT transporter (5-HTT) has significant skeletal effects [32]. Although the effect of the other genes in OP has not been reported, their functions in several diseases have been better understood. For example, it has been reported that LY9 antibody targeting depletes marginal zone and germinal center B cells in lymphoid tissues and reduces salivary gland inflammation in a mouse model of Sjögren's Syndrome [33]. Schmäh et al. found that the expression of P2RY8/CRLF2 was significantly upregulated in childhood with acute lymphoblastic leukemia, and suggested that it might be related to the development of acute lymphoblastic leukemia in childhood [34]. Previous studies have revealed that a compound heterozygous missense mutation and a large deletion in the KCTD7 gene presenting as an opsoclonus-myoclonus ataxia-like syndrome [35]. In addition, Liu et al. demonstrated that the inhibition of ITK can induce anti-tumor activity by downregulating TCR signaling pathway in malignant T cell lymphoma both *in vitro* and *in vivo* [36]. All these researches indicated that these 9 optimal genes might be involved in the occurrence and development of human diseases including OP. Therefore, a diagnostic model was established based on these 9 genes and verified that it could efficiently separate patients with or without OP. Moreover, the 5-fold cross validation method was conducted to evaluate the reliability of this model and indicated that the model had certain practical values.

However, more samples are needed to be collected to evaluate the reliability of our predictive model. In addition, although the role of several key genes has been well studied in OP progression, the others should be explored in detail in the future.

## Conclusion

In summary, a reliable diagnostic model was established based on 9 potential key genes including LCK, LY9, CD5, P2RY8, KCTD7, MDN1, ITK, CAPN2, and HTT, and we verified it could reliably separate patients with or without OP. Our results suggested that the diagnostic model might be potentially applied for the clinical diagnosis of OP.

## Supplementary Information

**Additional file 1.** Fig. S1 The distribution of mRNA expression values in each sample after GSE152073 standardization. The horizontal axis represents the samples and the vertical axis represent the relative expression of mRNA.

**Additional file 2.** Fig. S2 The diagnosis diagram of logistic regression model. (A) The normal Q-Q diagram of logistic regression model. The points should fall on a line at an angle of 45 degrees. If the deviation is too large, the model violates the normal assumption. (B) The diagram of Residuals vs. Leverage. The red dotted line indicates the COOK distance. Generally, a point with the COOK greater than 0.5 is a very "influentia" point, which affects the reliability of the model.

**Additional file 3.** Table S1. The full list of significantly enriched GO terms and KEGG pathways based on the 176 genes of the green module.

**Additional file 4.** Table S2. The top 50 genes screened from PPI network and respective scores.

## Data Availability

The datasets generated and analyzed during the current study are available in the [GEO] repository (https://www.ncbi.nlm.nih.gov/geo/).

## Abbreviations

OP: Osteoporosis
WGCNA: Weighted gene co-expression network analysis
PPI: Protein–protein interaction
BMD: Bone mineral density
GEO: Gene Expression Omnibus
ME: Module Eigengene
GO: Gene Ontology
KEGG: Kyoto Encyclopedia of Genes and Genomes
MNC: Maximum neighborhood component
WHO: World Health Organization
