# Supplementary figures and images for: Machine learning analysis of gene expression profile reveals a novel diagnostic signature for osteoporosis
Source: J Orthop Surg Res. 2021 Mar 15;16:189. doi: 10.1186/s13018-021-02329-1 (PMC7958453; doi:10.1186/s13018-021-02329-1)

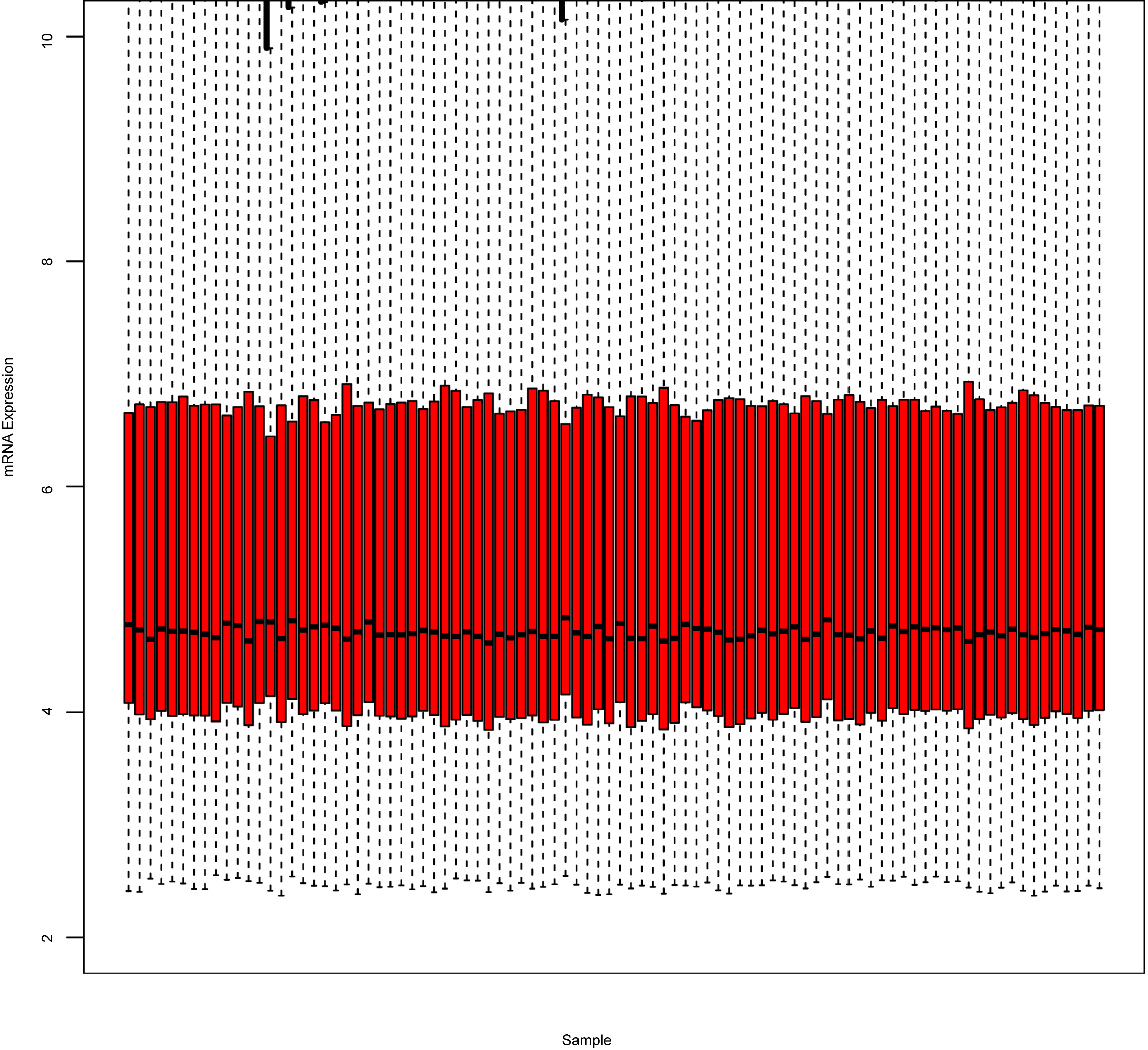

Supplement: Supplementary file 1 — Additional file 1. Fig. S1 The distribution of mRNA expression values in each sample after GSE152073 standardization. The horizontal axis represents the samples and the vertical axis represent the relative expression of mRNA. [file 13018_2021_2329_MOESM1_ESM.tif]

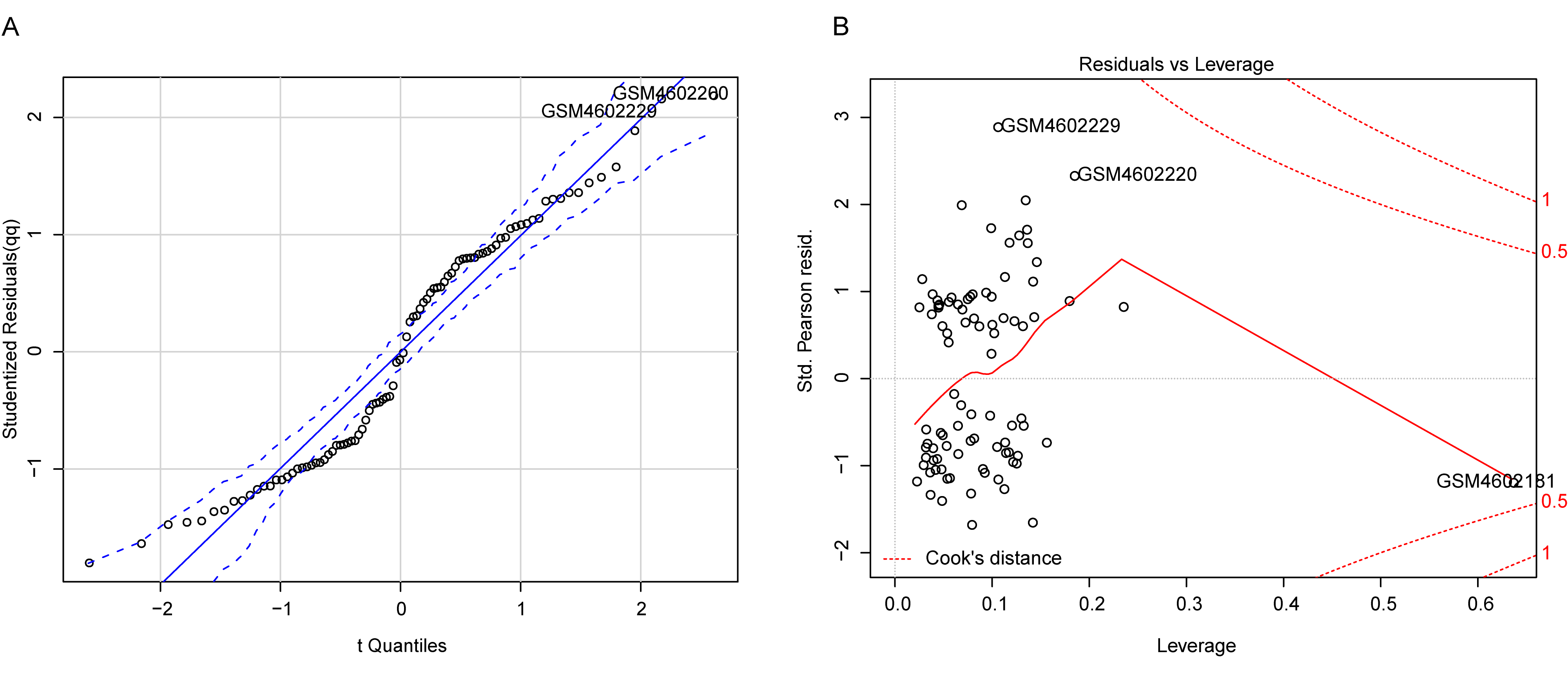

Supplement: Supplementary file 2 — Additional file 2. Fig. S2 The diagnosis diagram of logistic regression model. (A) The normal Q-Q diagram of logistic regression model. The points should fall on a line at an angle of 45 degrees. If the deviation is too large, the model violates the normal assumption. (B) The diagram of Residuals vs. Leverage. The red dotted line indicates the COOK distance. Generally, a point with the COOK greater than 0.5 is a very "influentia" point, which affects the reliability of the model. [file 13018_2021_2329_MOESM2_ESM.tif]
